# Synthesis and Broad-Spectrum Antiviral Activity of Some Novel Benzo-Heterocyclic Amine Compounds

**DOI:** 10.3390/molecules19010925

**Published:** 2014-01-15

**Authors:** Da-Jun Zhang, Wen-Fang Sun, Zhao-Jin Zhong, Rong-Mei Gao, Hong Yi, Yu-Huan Li, Zong-Gen Peng, Zhuo-Rong Li

**Affiliations:** Institute of Medicinal Biotechnology, Chinese Academy of Medical Sciences & Peking Union Medical College, Beijing 100050, China

**Keywords:** unsaturated five-membered benzo-heterocyclic amines, broad-spectrum antiviral activity, DNA and RNA virus, SAR

## Abstract

A series of novel unsaturated five-membered benzo-heterocyclic amine derivatives were synthesized and assayed to determine their *in vitro* broad-spectrum antiviral activities. The biological results showed that most of our synthesized compounds exhibited potent broad-spectrum antiviral activity. Notably, compounds **3f** (IC_50_ = 3.21–5.06 μM) and **3g** (IC_50_ = 0.71–34.87 μM) showed potent activity towards both RNA viruses (influenza A, HCV and Cox B3 virus) and a DNA virus (HBV) at low micromolar concentrations. An SAR study showed that electron-withdrawing substituents located on the aromatic or heteroaromatic ring favored antiviral activity towards RNA viruses.

## 1. Introduction

Viral infections pose a threat to virtually every organism in every domain of life. People over 65 years of age account for 90% of seasonal influenza-associated deaths, and influenza viruses with either high mortality or morbidity have heightened fears that the next influenza pandemic will occur soon [1,2]. The hepatitis C virus (HCV) is a significant bloodborne human pathogen that affects an estimated 3% of the world’s population, 80% of whom progress to a chronic infectious state [3,4]. Currently, the standard of care (SOC) is a combination of pegylated interferon and ribavirin, but this regimen can cause severe side effects and poorly sustained viral response [5,6]. Although two protease inhibitors (Victrelis (boceprevir) and Incivek (telaprevir)) were approved by the USA FDA in 2011, both must be used in combination with ribavirin and pegylated interferon, and there is still an unmet need for the treatment of HCV infections. Hepatitis B virus (HBV) is a hepatotropic noncytopathic envelope virus that causes acute and chronic hepatitis. Although a vaccine has been developed, chronic hepatitis B (CHB) caused by hepatitis virus infection is still one of the most serious human viral infectious diseases worldwide. Worldwide more than 350 million people suffer from chronic HBV infections, many of whom subsequently develop more severe liver diseases such as cirrhosis and hepatocellular carcinoma (HCC) [7]. Coxsackie B viruses are single-strand RNA viruses, and infection with Cox B can cause fever, headache, chest pain and other problems. Cardiac infection with Cox B3 can result in acute myocarditis that is spontaneously resolved or chronic myocarditis with prolonged viral persistence [8]. Currently, there is no specific treatment or vaccine available for Coxsackie virus infections.

Most current antiviral drugs, including those in development, are direct-acting antiviral (DAA) molecules that specifically target viral proteins. These drugs are narrow in spectrum and are vulnerable to the rapid emergence of viral resistance [9]. The emergence of drug-resistant viruses, especially multidrug-resistant strains, represents a significant problem in current clinical practice that needs to be addressed and should be considered a high priority for new avenues of research [10,11]. To fulfill all of these requirements, novel classes of antivirals are needed [12]. Additionally, due to the high mutation rates that are particularly prevalent in RNA viruses, the lifetime of specific antiviral therapeutics is often severely limited. Broad spectrum antivirals would be one way of circumventing this problem [13]. Consequently, it is highly desirable to generate these types of drugs, and scientists are encouraged to research and develop novel broad spectrum antivirals.

**Figure 1:**
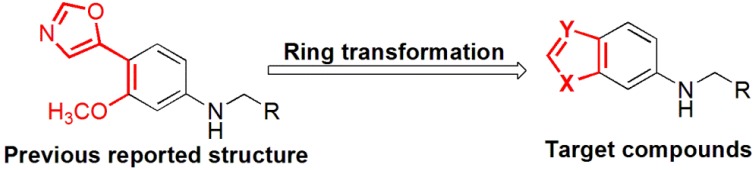
The chemical structure of the designed target compounds.

Previously, we have reported a novel class of (5-oxazolyl)phenyl amines that act as potential inosine monophosphate dehydrogenase (IMPDH) inhibitors and exhibit broad-spectrum antiviral activities [14]. In continuance of our endeavor to search for novel antiviral agents with broad-spectrum antiviral activity, we designed a novel class of unsaturated five-membered benzo-heterocyclic derivatives using a ring transforming strategy that is based on our previously reported structures (Figure 1).

Moreover, the secondary amine derivatives showed potent antiviral activity against CoxB3, CoxB6 and HCV in our previous study. Therefore, the secondary amine derivatives were primarily synthesized in this work. Additionally, unsaturated five-membered benzo-heterocyclic scaffolds appear to be privileged structures, which were widely distributed among bioactive compounds and successful drugs [15,16,17,18]. Consequently, a novel class of unsaturated five-membered benzo-heterocycle-substituted amines were synthesized and assayed for their broad spectrum antiviral activity *in vitro*, and their structure-activity relationships (SARs) were also investigated.

**Scheme 1:**
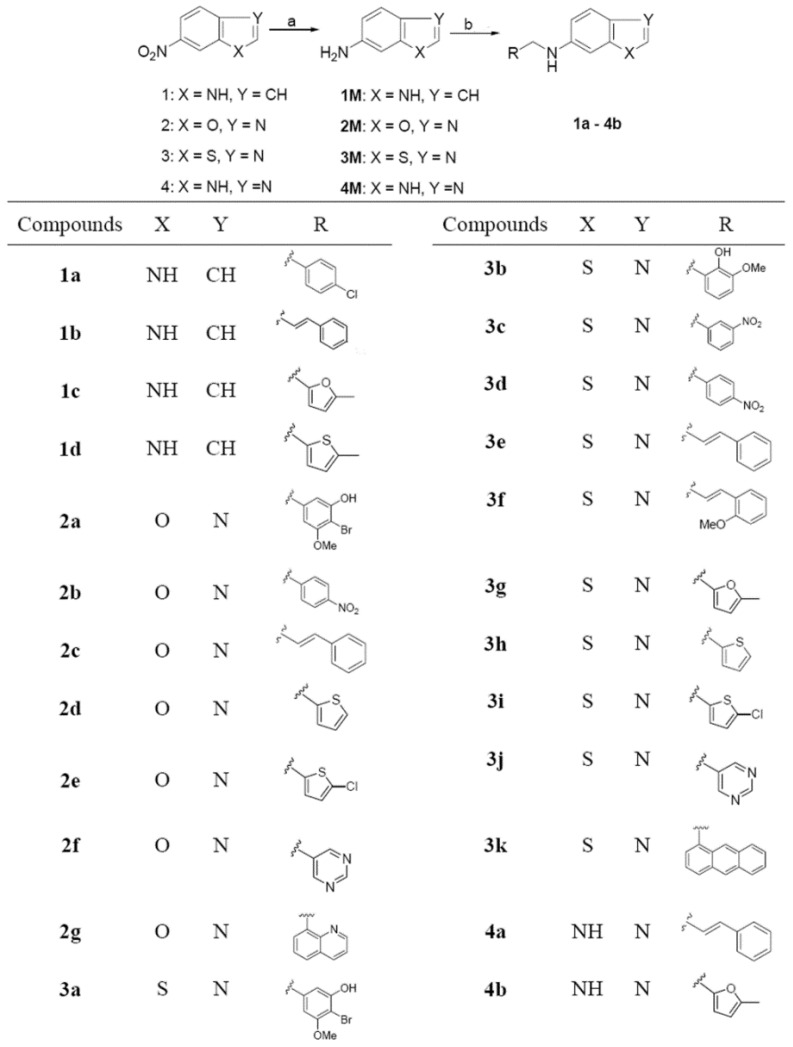
Synthetic route to the target compounds **1a**–**4b**.

*Reagents and conditions*: (a) 10% Pd/C, H_2_(50 psi), EtOH, rt; and (b) various substituted aldehydes, EtOH, rt or reflux, 8 h, then NaBH_4_, rt, 3 h.

## 2. Results and Discussion

### 2.1. Chemistry

As depicted in Scheme 1, a series of novel benzo-heterocyclic amine derivatives containing the substituted or non-substituted aromatic/heteroaromatic rings were synthesized. The commercially available compounds **1**–**4** were chosen as starting materials and were reduced at 50 psi hydrogen over Pd/C (10%) to produce the intermediate compounds **1M**–**4M** which have been reported in the literature [19,20,21,22]. The target compounds were obtained via reductive amination with a yield of 50%–65%. Specifically, compounds **1M**–**4M** were condensed with commercially available substituted aldehydes at room temperature or under reflux in ethanol to produce the intermediate Schiff bases, which were directly reduced without further purification in the presence of NaBH_4_ to generate the target compounds. In the case of compounds **4a** and **4b**, which were insoluble in methylene chloride, ethyl acetate was used to extract the target products after workup. The chemical structures of the synthesized target compounds were all confirmed by ^1^H-NMR, ^13^C-NMR and HRMS.

### 2.2. Biological Evaluation

The synthesized target compounds were assayed for their broad spectrum antiviral activity towards influenza A, HBV, HCV and Cox B3 virus *in vitro*. Specifically, the activity against influenza A and Cox B3 was determined by the cytopathic effect (CPE) inhibitory assay, and oseltamivir and ribavirin (RBV) were used as positive controls, respectively. The inhibitory activities towards HBV and HCV were tested by real time PCR, and lamivudine (3TC) and teleprevir were used as positive controls, respectively. The results are summarized in Table 1.

In general, the majority of our synthesized compounds showed definitive broad spectrum activity against the viruses tested especially compounds **3f** and **3g**, which exhibited potent activity towards the entire virus panel tested. IC_50_ values ranged from 0.71 μM to 34.87 μM. Additionally, most of our synthesized compounds showed potent inhibitory activity towards Influenza A, HCV and Cox B3 virus simultaneously, including compounds **2c**,**e**,**g**, **3d**–**g**, **3i** and **4a**. However, only 3 compounds (**1a**, **3f** and **3g**) showed inhibitory activity toward HBV. Based on these results, we concluded that our synthesized compounds were more sensitive to RNA viruses (Influenza A, HCV and Cox B3) when compared to a DNA virus (HBV).

#### 2.2.1. Anti-influenza Virus Activity

The majority of the synthesized compounds exhibited potent antiviral activity towards influenza A/hanfang/359/95 virus. This was particularly true for compounds **1d**, **2c**,**e**,**g**, **3b**–**f**,**h**,**i** and **4a** because the IC_50_ values were superior or comparable to the positive control drugs (RBV and oseltamivir). Notably, compound **3d** showed both potent inhibitory activity against influenza A (IC_50_ = 3.36 μM) and the highest SI value (80.8) among the entire panel of synthesized compounds.

With regard to the structure-activity relationships, the activity of compounds **4a**,**b**, and **3e**,**g** were superior to their indole and benzoxazole scaffold counterparts; we concluded that the benzimidazole and benzothiazole moieties favored anti-influenza activity. Replacement of the hydrogen at the 4 position of the thiophene ring (**2d** and **3h**) with a chloro group (**2e** and **3i**) increased the compounds’ anti-influenza activity. The derivatives **2b** and **3d** containing a strong electron withdrawing group (NO_2_) substituted at the *para*-position of the benzene ring also showed potent inhibitory activity. Migration of the NO_2_ group from the *para*-position (**3d**) to the *meta*-position (**3c**) slightly decreased the activity. Based on these results, we concluded that an electron withdrawing substituent on the aromatic or heteroaromatic ring favored anti-influenza A activity.

**Table 1:** The antiviral activity and cytotoxicity of the compounds.

NO.	Influenza A/hanfang/359/95			Cox B3			HCV			HBV		
	TC_50_ (μM)	IC_50_ (μM)	SI	TC_50_ (μM)	IC_50_ (μM)	SI	TC_50_ (μM)	IC_50_ (μM)	SI	TC_50_ (μM)	IC_50_ (μM)	SI
**1a**	3.35	>1.60	-	8.34	4.79	1.7	15.88	1.40	11.3	8.02	1.44	5.6
**1b**	2.86	>1.65	-	8.61	2.86	3.0	NT	NT	NT	3.21	>1.85	-
**1c**	4.52	>0.66	-	25.15	1.93	13.0	46.78	2.77	16.8	16.67	>5.56	-
**1d**	26.50	11.84	2.2	66.10	1.69	39.1	NT	NT	NT	28.87	>16.67	-
**2a**	64.04	>10.60	-	55.13	3.52	15.7	89.63	32.95	2.7	28.87	>16.67	-
**2b**	288.23	41.26	7.0	257.52	31.34	8.2	>200	>66.67	<3.0	16.67	>5.56	-
**2c**	133.16	11.59	11.5	103.36	1.24	83.5	105.44	10.48	10.1	28.87	>16.67	-
**2d**	434.24	34.43	11.6	100.35	9.29	10.8	136.53	>66.67	<2.0	NT	NT	NT
**2e**	87.30	10.95	8.0	29.08	3.55	8.2	45.78	17.72	2.6	19.25	>11.11	-
**2f**	425.44	>98.32	-	295.10	56.73	5.2	NT	NT	NT	NT	NT	NT
**2g**	56.04	9.04	6.2	80.80	8.98	9.0	27.57	14.18	1.9	NT	NT	NT
**3a**	21.08	>10.13	-	21.08	10.13	2.1	108.82	48.99	2.2	28.87	>16.67	-
**3b**	55.95	10.02	5.6	67.23	5.66	11.9	126.59	38.01	3.3	57.74	>33.33	-
**3c**	45.01	12.47	3.6	112.45	7.23	15.6	NT	NT	NT	NT	NT	NT
**3d**	272.01	3.36	80.8	181.3	1.82	99.5	142.86	22.50	6.3	9.62	>5.56	-
**3e**	24.10	8.60	2.8	20.05	1.16	17.2	43.58	9.75	4.5	9.62	>5.56	-
**3f**	21.66	4.15	5.2	12.48	3.21	3.9	54.99	5.04	10.9	28.87	5.06	5.7
**3g**	94.60	26.24	3.6	65.57	5.03	13.0	138.38	34.87	4.5	21.48	0.71	30.3
**3h**	78.14	6.58	11.9	65.03	10.43	6.2	153.72	47.64	3.2	NT	NT	NT
**3i**	27.42	3.42	8.0	13.18	2.53	5.2	38.24	9.81	3.9	9.62	>5.56	-
**3j**	826.44	213.80	3.9	826.44	52.98	15.6	>200	>66.67	<3.0	28.87	>16.67	-
**3k**	1.73	>0.41	-	2.09	-	-	NT	NT	NT	NT	NT	NT
**4a**	14.84	4.93	3.0	25.75	6.38	4.0	79.04	5.51	14.3	NT	NT	NT
**4b**	440.02	21.43	20.5	254.07	48.89	5.2	>200	24.37	>8.2	>50	>50	-
**RBV**	4765.44	8.76	544.0	8205.90	2120.4	3.9	NT	NT	NT	NT	NT	NT
**Oseltamivir**	4030.42	18.97	212.5	NT	NT	NT	NT	NT	NT	NT	NT	NT
**3TC**	NT	NT	NT	NT	NT	NT	NT	NT	NT	>100	1.85	>54.1
**Telaprevir**	NT	NT	NT	NT	NT	NT	32.12	0.011	2920.0	NT	NT	NT

TC_50_, 50% cytotoxic concentration; IC_50_, 50% inhibition concentration; SI, the selectivity index. NT means not tested.

However, in the case of compound **3e**, introduction of an electron-donating methoxyl group at the *ortho*-position of the benzene ring (**3f**) also increased antiviral activity, and this effect may not be due to its electron-donating property, but rather to the hydrogen bond-accepting properties of the methoxyl group. A complete loss in activity of compound **3k** containing a bulky anthracene group may be due to steric hindrance.

#### 2.2.2. Anti Cox B3 Virus Activity

As shown in Table 1, all of the synthesized compounds except **3k** showed potent inhibitory activity towards Cox B3 at low micromolar concentrations, especially compounds **1a**–**d**, **2a**,**c**,**e**, **3d**–**f** and **3i**, and the IC_50_ values were less than 5.0 μM. Notably, compounds **3d** and **2c** exhibited relatively high SI values (99.5 and 83.5, respectively). Based on the activity of compounds **1b**, **2c**, **3e** and **4a**, we concluded that only the benzimidazole slightly disfavored anti-Cox B3 activity, and there was no significant difference among the other three scaffolds. Similar to the anti-influenza activity SAR, the derivatives containing electron-withdrawing groups (**2e**, **3d** and **3i**) showed relatively more potent activity. Substitution of an electron-donating methoxyl group (**3f**) for the hydrogen atom at the *ortho-* position of the benzene ring (**3e**) disfavored antiviral activity. Migration of the NO_2_ group from the para position (**3d**) to the *meta*-position (**3c**) also decreased its activity. Compound **3k** showed no inhibitory activity, likely due to high steric hindrance.

#### 2.2.3. Anti HCV Activity

As shown in Table 1, compounds **1a**,**c**, **3e**,**f**,**i** and **4a** showed relatively potent anti-HCV activity with IC_50_ values of less than 10.0 μM. The IC_50_ values of all of the synthesized compounds were inferior to the positive control telaprevir, an NS3/4A protease inhibitor. However, the synthesized compounds exhibited less cytotoxicity when compared to telaprevir. Based on the activity of compounds **1c**, **3g** and **4b**, the indole scaffold appeared to be the optimal fragment for anti-HCV activity. Given that compounds **2e** and **3i**, which contain chloro groups at the thiophene ring, were more potent that their non-substituted counterparts, we concluded that the electron-withdrawing substituents were favorable for anti-HCV activity, a result that is consistent with the SAR of both anti-influenza A and anti-Cox B3 activities. The most potent of the synthesized compounds was also the compound containing a chloro group at the *para*-position of the benzene ring (**1a**). The derivatives containing a styryl substituent (**2c**, **3e**, **3f** and **4a**) also showed potent anti-HCV activity at low micromolar concentrations.

#### 2.2.4. Anti-HBV Activity

HBV was less sensitive to the synthesized compounds compared to influenza A, Cox B3 and HCV virus, and only compounds **1a**, **3f** and **3g** showed definitive activity towards HBV. Among these, compounds **1a** and **3g** were more potent than the positive control 3TC, especially compound **3g** which exhibited an IC_50_ value of less than 1.0 μM (0.7 μM). Some simple SARs can be determined based on the biological results. Although compound **3f** showed potent inhibitory activity towards HBV, compound **3e** which lacks a methoxyl group at the *ortho*-position of the benzene ring did not exhibit any activity. Hence, the methoxyl group was indispensable for the anti-HBV activity of compound **3f**. Additionally, substitution of the indole (**1c**) or benzimidazole (**4b**) scaffold for the benzothiazole (**3g**) fragment led to a complete loss of antiviral activity.

## 3. Experimental

### 3.1. General Information

All solvents and reagents were of chemical or analytical grade. The progress of the reactions was monitored by TLC using solvent systems of different polarities. ^1^H-NMR and ^13^C-NMR spectra were recorded with a BrukerBioSpin GmbH (Rheinstetten, Germany) spectrometer at 400 or 500 MHz. Chemical shifts are reported in parts per million relative to a tetramethylsilane internal standard. Melting points were determined with an X6 microscope melting point apparatus and were not corrected. All mass spectra (MS, HRMS) were recorded on a LTQ-Orbitrap linear ion trap high-resolution mass spectrometer (ThermoFisher Scientific, San Jose, CA, USA).

### 3.2. Chemistry

#### 3.2.1. General procedure for the synthesis of **1M**–**4M**

A mixture of compounds **1**–**4** (6.13 mmol) in ethanol (30 mL) was treated with 10% Pd/C (20 wt. % of **1**–**4**) and subjected overnight to 50 psi H_2_ (g) in a Parr hydrogenation apparatus. The reaction mixture was filtered and concentrated *in vacuo*. Pure products **1M**–**4M** were obtained via flash chromatography by eluting with a gradient of 30%–40% EtOAc/hexanes.

*1H-Indol-6-ylamine* (**1M**). Gray solid, mp: 67–68 °C; 90% yield; ^1^H-NMR (400 MHz, CDCl_3_) δ (ppm): 7.87 (1H, brs), 7.40 (1H, d, *J* = 8.4 Hz), 7.00 (1H, m), 6.67(1H,s), 6.57 (1H, dd, *J*_1_ = 8.4 Hz, *J*_2_ = 2.0 Hz), 6.42 (1H, m).

*Benzooxazol-6-ylamine* (**2M**). Yellow solid, mp: 86–87 °C; 86% yield; ^1^H-NMR (400 MHz, DMSO-*d*_6_) δ (ppm): 8.32 (1H, s), 7.39 (1H, d, *J* = 8.8 Hz), 6.78 (1H, d, *J* = 2.0 Hz), 6.64 (1H, dd, *J*_1_ = 8.8 Hz, *J*_2_ = 2.0 Hz), 5.36 (1H, br).

*Benzothiazol-6-ylamine* (**3M**). Yellow solid, mp: 88–89 °C; 88% yield; ^1^H-NMR (400 MHz, DMSO-*d*_6_) δ (ppm): 8.88 (1H, s), 7.71 (1H, d, *J* = 8.4 Hz), 7.12 (1H, d, *J* = 2.0 Hz), 6.81 (1H, dd, *J*_1_ = 8.4 Hz, *J*_2_ = 2.0 Hz), 5.40 (2H, br).

*3H-Benzoimidazol-5-ylamine* (**4M**). Brown solid, mp: 121–122 °C; 85% yield; ^1^H-NMR (400 MHz, DMSO-*d*_6_) δ (ppm): 7.85 (1H, s), 7.24 (1H, d, *J* = 8.4 Hz), 6.66 (1H, s), 6.50 (1H, dd, *J*_1_ = 8.4 Hz, *J*_2_ = 2.0 Hz), 4.80 (2H, br).

#### 3.2.2. General procedure for the synthesis of **1a**–**1e**, **2a**–**2g**, **3a**–**3f**

A mixture of the appropriate amine (**1M**–**3M**, 2 mmol) and the appropriate aldehyde (2.2 mmol) in ethanol (10 mL) was stirred at room temperature for 8 h and NaBH_4_ (2.2 mmol) was subsequently added to the solution. After 3 h, the reaction was completed. Water (5 mL) was then added to the solution and neutralized with aqueous 10% HCl. CH_2_Cl_2_ (40 mL) was added to the solution, and the organic layer was separated, washed with saturated aqueous sodium chloride and subsequently dried over anhydrous Na_2_SO_4_. The solvent was removed under a vacuum. The residue was purified by chromatography on silica gel (EtOAc/*n*-hexane = 1/3) to generate the corresponding compounds.

Characterization data for the title compounds: 

*(4-Chlorobenzyl)-(1H-indol-6-yl)-amine* (**1a**). White solid, mp: 135–137 °C, 50% yield; ^1^H-NMR (DMSO-*d*_6_, 500 MHz) δ (ppm): 10.42 (1H, s), 7.39–7.31 (4H, m), 7.20–7.18 (1H, m), 6.93–6.92 (1H, m), 6.47–6.45 (1H, m), 6.35 (1H, s), 6.15 (1H, s), 5.93 (1H, t, *J* = 6.0 Hz), 4.26 (2H, d, *J* = 6.0 Hz). ^13^C-NMR (125 MHz, DMSO-*d*_6_) δ (ppm): 144.6, 140.4, 137.8, 130.4, 129.4 (2C), 128.6 (2C), 122.2, 120.7, 119.9, 109.7, 101.3, 93.3, 47.1. HR-MS (ESI^+^) *m/z*: 257.0844 [M+H]^+^, calcd for C_15_H_14_ClN_2_^+^, 257.0845.

*(E)-(1H-Indol-6-yl)-(3-phenylallyl)-amine* (**1b**). White solid, mp: 130–132 °C, 53% yield; ^1^H-NMR (500 MHz, DMSO-*d*_6_) δ (ppm): 10.47 (1H, s), 7.40–7.39 (2H, m),7.32–7.29 (2H, m), 7.22–7.19 (2H, m), 6.96–6.95 (1H, m), 6.61 (1H, d, *J* = 16.0 Hz), 6.51 (1H, s), 6.47 (1H, dd, *J*_1_ = 8.5 Hz, *J*_2_ = 2.0 Hz), 6.43–6.41 (1H, m), 6.17 (1H, s), 5.52 (1H, t, *J* = 5.5 Hz), 3.86–3.84 (2H, m). ^13^C-NMR (125 MHz, DMSO-*d*_6_) δ (ppm): 145.0, 138.0, 137.4, 130.2, 129.3, 129.2 (2C), 127.7, 126.5 (2C), 122.2, 120.6, 119.9, 109.7, 101.3, 93.2, 46.3. HR-MS (ESI^+^) *m/z*: 249.1390 [M+H]^+^, calcd for C_17_H_17_N_2_^+^, 249.1392.

*(1H-Indol-6-yl)-(5-methylfuran-2-ylmethyl)-amine* (**1c**). White solid, mp: 100–102 °C, 50% yield; ^1^H-NMR (400 MHz, DMSO-*d*_6_) δ (ppm): 10.49 (1H, s), 7.19 (1H, d, *J* = 8.4 Hz), 6.96–6.95 (1H, m), 6.52 (1H, s), 6.46 (1H, dd, *J*_1_ = 8.4 Hz, *J*_2_ = 2.0 Hz), 6.17–6.16 (1H, m), 6.12 (1H, d, *J* = 2.8 Hz), 5.95 (1H, d, *J* = 2.0 Hz), 5.63 (1H, t, *J* = 6.0 Hz), 4.15 (2H, d, *J* = 6.0 Hz), 2.22 (3H, s). ^13^C-NMR (125 MHz DMSO-*d*_6_) δ (ppm): 152.4, 150.7, 144.6, 137.9, 122.3, 120.6, 120.0, 109.6, 107.9, 106.7, 101.3, 93.3, 41.5, 13.8. HR-MS(ESI^+^) *m/z*: 227.1185 [M+H]^+^, calcd for C_14_H_15_N_2_O^+^, 227.1184.

*(1H-Indol-6-yl)-(5-methylthiophen-2-ylmethyl)-amine* (**1d**). White solid, mp: 89–91 °C, 50% yield; ^1^H-NMR (500 MHz, DMSO-*d*_6_) δ (ppm): 10.47 (1H, s), 7.19 (1H, d, *J* = 8.5 Hz),6.96–6.95 (1H, m), 6.79 (1H, d, *J* = 3.0 Hz), 6.60 (1H, d, *J* = 2.0 Hz), 6.51 (1H, s), 6.47 (1H, dd, *J*_1_ = 8.5 Hz, *J*_2_ = 2.0 Hz), 6.17 (1H, s), 5.78 (1H, t, *J* = 6.0 Hz), 4.33 (2H, d, *J* = 6.0 Hz), 2.35 (3H, s). ^13^C-NMR (125 MHz DMSO-*d*_6_) δ (ppm): 144.5, 143.1, 137.9, 137.8, 125.2, 124.6, 122.3, 120.6, 120.1, 109.8, 101.3, 93.6, 43.6, 15.5. HR-MS(ESI^+^) *m/z*: 243.0954 [M+H]^+^, calcd for C_14_H_15_N_2_S^+^, 243.0956.

*4-(Benzooxazol-6-ylaminomethyl)-2-bromo-6-methoxyphenol* (**2a**). White solid, mp: 129–131 °C, 53% yield; ^1^H-NMR (400 MHz, DMSO-*d*_6_) δ (ppm): 9.27 (1H, s), 8.33 (1H, s), 7.42 (1H, d, *J* = 8.4 Hz), 7.08 (1H, s), 7.01 (1H, s), 6.76 (1H, d, *J* = 2.0 Hz), 6.72 (1H, dd, *J*_1_ = 8.4 Hz, *J*_2_ = 2.0 Hz), 6.51 (1H, t, *J* = 6.0 Hz), 4.20 (2H, d, *J* = 6.0 Hz), 3.80 (3H, s).^13^C-NMR (125 MHz DMSO-*d*_6_) δ (ppm): 151.6, 151.4, 148.9, 148.2, 142.9, 132.2, 130.5, 123.2, 120.3, 112.4, 111.1, 109.7, 92.9, 56.6, 46.6. HR-MS(ESI^+^) *m/z*: 349.0178 [M+H]^+^, calcd for C_15_H_14_N_2_O_3_Br^+^, 349.0188.

*Benzooxazol-6-yl-(4-nitrobenzyl)-amine* (**2b**). Brown solid, mp: 154–156 °C, 65% yield; ^1^H-NMR (400 MHz, DMSO-*d*_6_) δ (ppm): 8.33 (1H, s), 8.19 (2H, d, *J* = 8.8 Hz), 7.64 (2H, d, *J* = 8.8 Hz), 7.45–7.43 (1H, d, *J* = 8.4 Hz), 6.80 (1H, t, *J* = 6.0 Hz), 6.73–6.72 (2H, m), 4.49 (2H, d, *J* = 6.0 Hz). ^13^C-NMR (125 MHz DMSO-*d*_6_) δ (ppm): 151.6, 151.5, 149.0, 147.9, 147.0, 130.7, 128.7 (2C), 124.0 (2C), 120.5, 112.3, 93.0, 46.6. HR-MS (ESI^+^) *m/z*: 270.0879 [M+H]^+^, calcd for C_14_H_12_N_3_O_3_^+^, 270.0879.

*(E)-Benzooxazol-6-yl-(3-phenylallyl)-amine* (**2c**). Light green solid, mp: 100–103 °C, 55% yield; ^1^H-NMR (400 MHz, DMSO-*d*_6_) δ (ppm): 8.34 (1H, s), 7.45–7.40 (3H, m), 7.33–7.29 (2H,m), 7.24–7.20 (1H,m), 6.82 (1H, d, *J* = 2.0 Hz), 6.74 (1H, dd, *J*_1_ = 8.8 Hz, *J*_2_ = 2.0 Hz), 6.63 (1H, d, *J* = 16.0 Hz), 6.38 (1H, m), 6.27(1H, t, *J* = 5.6 Hz), 3.91–3.87 (2H, m). ^13^C-NMR (125 MHz DMSO-*d*_6_) δ (ppm): 151.7, 151.3, 148.5, 137.2, 130.8, 129.1 (2C), 128.9, 128.1, 127.8, 126.6 (2C), 120.2, 112.3, 92.7, 45.7. HR-MS (ESI^+^) *m/z*: 251.1176 [M+H]^+^, calcd for C_16_H_15_N_2_O^+^, 251.1184.

*Benzooxazol-6-yl-thiophen-2-ylmethylamine* (**2d**). Light green solid, mp: 62–64 °C, 62% yield; ^1^H-NMR (400 MHz, DMSO-*d*_6_) δ (ppm): 8.35 (1H, s), 7.43 (1H, d, *J* = 8.8 Hz), 7.37 (1H, d, *J* = 5.2 Hz), 7.09 (1H, d, *J* = 3.2 Hz), 6.98–6.95 (1H, m), 6.85 (1H, d, *J* = 2.0 Hz), 6.75 (1H, dd, *J*_1_ = 8.8 Hz, *J*_2_ = 2.0 Hz), 6.59 (1H, t, *J* = 6.0 Hz), 4.50 (2H, d, *J* = 6.0 Hz). ^13^C-NMR (125 MHz DMSO-*d*_6_): δ (ppm): 151.6, 151.5, 148.0, 144.2, 130.6, 127.3, 125.6, 125.1, 120.3, 112.5, 93.1, 42.7. HR-MS (ESI^+^) *m/z*: 231.0582 [M+H]^+^, calcd for C_12_H_11_N_2_OS^+^, 231.0592.

*Benzooxazol-6-yl-(5-chlorothiophen-2-ylmethyl)-amine* (**2e**). Light green solid, mp: 100–102 °C, 58% yield; ^1^H-NMR (400 MHz, DMSO-*d*_6_) δ (ppm): 8.36 (1H, s), 7.45 (1H, d, *J* = 8.8 Hz), 6.97–6.95 (2H, m), 6.86 (1H, d, *J* = 2.0 Hz), 6.73 (1H, dd, *J*_1_ = 8.8 Hz, *J*_2_ = 2.0 Hz), 6.63 (1H, t, *J* = 6.0 Hz), 4.45 (2H, *J* = 6.0 Hz). ^13^C-NMR (125 MHz DMSO-*d*_6_) δ (ppm): 151.7, 151.5, 147.7, 144.2, 130.9, 126.9, 126.7, 125.3, 120.1, 112.6, 93.3, 42.9. HR-MS (ESI^+^) *m/z*: 265.0210 [M+H]^+^, calcd for C_12_H_10_N_2_OSCl^+^, 265.0202.

*Benzooxazol-6-yl-pyrimidin-5-ylmethyl-amine* (**2f**). Gray solid, mp: 175–177 °C, 51% yield; ^1^H-NMR (500 MHz, DMSO-*d*_6_) δ (ppm): 9.06 (1H, s), 8.81 (2H, s), 8.35 (1H, s), 7.45 (1H, d, *J* = 8.5 Hz), 6.85 (1H, d, *J* = 1.5 Hz), 6.74 (1H, dd, *J*_1_ = 8.5 Hz, *J*_2_ = 1.5 Hz), 6.61 (1H, t, *J* = 6.0 Hz), 4.38 (2H, d, *J* = 6.0 Hz). ^13^C-NMR (125 MHz DMSO-*d*_6_) δ (ppm): 157.6, 156.8 (2C), 156.0, 151.7, 151.6, 133.5, 130.8, 120.5, 112.5, 93.2, 42.6. HR-MS (ESI^+^) *m/z*: 227.0934 [M+H]^+^, calcd for C_12_H_11_N_4_O^+^, 227.0933.

*Benzooxazol-6-yl-quinolin-8-ylmethylamine* (**2g**). Gray solid, mp: 98–100 °C, 63% yield; ^1^H-NMR (500 MHz, DMSO-*d*_6_) δ (ppm): 8.99–8.98 (1H, m), 8.39(1H, d, *J* = 8.5 Hz), 8.29 (1H, s), 7.87 (1H, d, *J* = 8.0 Hz), 7.73 (1H, d, *J* = 7.0 Hz), 7.60–7.57 (1H, m), 7.56–7.52 (1H, m), 7.42–7.40 (1H, m), 6.75–6.74 (2H, m), 6.62 (1H, t, *J* = 6.0 Hz), 4.95 (2H, d, *J* = 6.0 Hz). ^13^C-NMR (125 MHz DMSO-*d*_6_) δ (ppm): 151.7, 151.3, 150.2, 148.6, 146.4, 137.4, 137.0, 130.4, 128.4, 127.9, 127.4, 126.8, 121.9, 120.3, 112.2, 92.7, 42.4. HR-MS (ESI^+^) *m/z*: 276.1125 [M+H]^+^, calcd for C_17_H_14_N_3_O^+^, 276.1137.

*4-(Benzothiazol-6-ylaminomethyl)-2-bromo-6-methoxyphenol* (**3a**). White solid, mp: 193–195 °C, 54% yield; ^1^H-NMR (400 MHz, DMSO-*d*_6_) δ (ppm): 9.27 (1H, s), 8.89 (1H, s), 7.54 (1H,d, *J* = 8.4 Hz), 7.10 (1H, d, *J* = 2.4 Hz), 7.08 (1H, s), 7.01 (1H, d, *J* = 2.4 Hz), 6.89 (1H, dd, *J*_1_ = 8.4 Hz, *J*_2_ = 2.4 Hz), 6.53 (1H, d, *J* = 6.0 Hz), 4.22 (2H, d, *J* = 6.0 Hz), 3.80 (3H, s).^13^C-NMR (125 MHz DMSO-*d*_6_) δ (ppm): 149.9, 148.9, 147.5, 145.4, 143.0, 135.9, 132.2, 123.5, 123.2, 115.0, 111.1, 109.7, 102.0, 56.6, 46.5. HR-MS (ESI^+^) *m/z*: 364.9953 [M+H]^+^, calcd for C_15_H_14_N_2_O_2_SBr^+^, 364.9959.

*2-(Benzothiazol-6-ylaminomethyl)-6-methoxyphenol* (**3b**). Pale yellow solid, mp: 206–208 °C, 59% yield; ^1^H-NMR (400 MHz, DMSO-*d*_6_) δ (ppm): 8.87 (1H, s), 8.73 (1H, s), 7.72 (1H, d, *J* = 8.8 Hz), 7.05 (1H, d, *J* = 2.0 Hz), 6.88 (1H, dd, *J*_1_ = 8.8 Hz, *J*_2_ = 2.0 Hz), 6.85 (1H, s), 6.83 (1H, s), 6.72–6.68 (1H, m), 6.38 (1H, t, *J* = 6.0 Hz), 4.26 (2H, d, *J* = 6.0 Hz), 3.80 (3H, s). ^13^C-NMR (125 MHz DMSO-*d*_6_) δ (ppm): 149.6, 147.9, 147.8, 145.2, 144.4, 135.9, 126.4, 123.5, 120.7, 119.1, 114.8, 110.9, 101.5, 56.3, 41.9. HR-MS (ESI^+^) *m/z*: 287.0851 [M+H]^+^, calcd for C_15_H_15_N_2_O_2_S^+^, 287.0854.

*Benzothiazol-6-yl-(3-nitrobenzyl)-amine* (**3c**). Yellow solid, mp: 126–128 °C, 54% yield; ^1^H-NMR (500 MHz, DMSO-*d*_6_) δ (ppm): 8.89 (1H, s), 8.24 (1H, s), 8.09 (1H, dd, *J*_1_ = 8.0 Hz, *J*_2_ = 1.5 Hz), 7.84 (1H, d, *J* = 8.0 Hz), 7.74 (1H, d, *J* = 9.0 Hz), 7.64–7.61 (1H, m), 7.11 (1H, d, *J* = 2.5 Hz), 6.90 (1H, dd, *J*_1_ = 9.0 Hz, *J*_2_ = 2.5 Hz), 6.80 (1H, t, *J* = 6.0 Hz), 4.49 (2H, d, *J* = 6.0 Hz). ^13^C-NMR (125 MHz DMSO-*d*_6_) δ (ppm): 150.2, 148.4, 147.1, 145.6, 143.2, 136.0, 134.4, 130.3, 123.7, 122.3, 122.2, 115.0, 102.1, 46.2. HR-MS (ESI^+^) *m/z*: 286.0661 [M+H]^+^, calcd for C_14_H_12_N_3_O_2_S^+^, 286.0650.

*Benzothiazol-6-yl-(4-nitrobenzyl)-amine* (**3d**). Brown solid, mp: 157–159 °C, 57% yield; ^1^H-NMR (400 MHz, DMSO-*d*_6_) δ (ppm): 8.90 (1H, s), 8.20 (2H, d, *J* = 8.4 Hz), 7.75 (1H, d, *J* = 8.8 Hz), 7.64 (2H, d, *J* = 8.4 Hz), 7.07 (1H, d, *J* = 2.4 Hz), 6.89 (1H, dd, *J*_1_ = 8.8 Hz, *J*_2_ = 2.4 Hz), 6.84–6.80 (1H, t, *J* = 6.4 Hz), 4.50 (2H, d, *J* = 6.4 Hz). ^13^C-NMR (125 MHz DMSO-*d*_6_) δ (ppm): 150.2, 149.0, 147.2, 147.0, 145.5, 136.0, 128.7 (2C), 124.0 (2C), 123.7, 114.9, 102.0, 46.5. HR-MS (ESI^+^) *m/z*: 286.0652 [M+H]^+^, calcd for C_14_H_12_N_3_O_2_S^+^, 286.0650.

*(E)-benzothiazol-6-yl-(3-phenyl-allyl)-amine* (**3e**). Brown solid, mp: 99–101 °C, 57% yield; ^1^H-NMR (400 MHz, DMSO-*d*_6_) δ (ppm): 8.89 (1H, s), 7.75 (1H, d, *J* = 8.8 Hz), 7.41 (2H, d, *J* = 7.6 Hz), 7.33–7.29 (2H,m), 7.24–7.20 (1H, m), 7.16 (1H, d, *J* = 2.4 Hz), 6.90 (1H, dd, *J*_1_ = 8.8 Hz, *J*_2_ = 2.0 Hz), 6.63 (1H, d, *J* = 16.0 Hz), 6.43–6.36 (1H, m), 6.30 (1H, t, *J* = 5.2 Hz), 2.50 (2H, t, *J* = 5.2 Hz). ^13^C-NMR (125 MHz DMSO-*d*_6_) δ (ppm): 149.8, 147.7, 145.3, 137.2, 136.0, 130.8, 129.1(2C), 128.1, 127.8, 126.6(2C), 123.5, 115.0, 101.7, 45.5. HR-MS (ESI^+^) *m/z*: 267.0956 [M+H]^+^, calcd for C_16_H_15_N_2_S^+^, 267.0956.

*(E)-benzothiazol-6-yl-[3-(2-methoxy-phenyl)-allyl]-amine* (**3f**). Pale yellow solid, mp: 107–109 °C, 60% yield; ^1^H-NMR (400 MHz, DMSO-*d*_6_) δ (ppm): 8.89 (1H, s), 7.75 (1H, d, *J* = 8.8 Hz), 7.46 (1H, dd, *J*_1_ = 7.6 Hz, *J*_2_ = 1.6 Hz), 7.24–7.20 (1H, m), 7.15 (1H, d, *J* = 2.4 Hz), 6.98 (1H, d, *J* = 8.8 Hz), 6.92–6.95 (3H, m), 6.38–6.28 (2H, m), 3.92–3.90 (2H, m), 3.77 (3H, s). ^13^C-NMR (125 MHz DMSO-*d*_6_) δ (ppm): 156.7, 149.7, 147.8, 145.2, 136.0, 129.1, 128.5, 127.0, 125.7, 125.6, 123.5, 121.0, 115.0, 111.8, 101.7, 55.8, 46.0. HR-MS (ESI^+^) *m/z*: 297.1060 [M+H]^+^, calcd for C_17_H_17_N_2_OS^+^, 297.1062.

*Benzothiazol-6-yl-(5-methylfuran-2-ylmethyl)-amine* (**3g**). Pale yellow solid, mp: 52–54 °C, 63% yield; ^1^H-NMR (400 MHz, DMSO-*d*_6_) δ (ppm): 8.90 (1H, s), 7.74 (1H, d, *J* = 8.8 Hz), 7.20 (1H, d, *J* = 2.0 Hz), 6.91 (1H, dd, *J*_1_ = 8.8 Hz, *J*_2_ = 2.0 Hz), 6.41 (1H, t, *J* = 6.0 Hz), 6.21 (1H, d, *J* = 2.8 Hz), 5.98–5.97 (1H, *J* = 2.8 Hz), 4.23 (2H, d, *J* = 6.0 Hz), 2.22 (3H, s). ^13^C-NMR (125 MHz DMSO-*d*_6_) δ (ppm): 151.4, 151.1, 149.6, 147.3, 145.4, 135.0, 123.5, 115.0, 108.2, 106.7, 101.9, 40.5, 13.8. HR-MS (ESI^+^) *m/z*: 245.0742 [M+H]^+^, calcd for C_13_H_13_N_2_OS^+^, 245.0749.

*Benzothiazol-6-yl-thiophen-2-ylmethylamine* (**3h**). Light brown solid, mp: 148–150 °C, 61% yield; ^1^H-NMR (400 MHz, DMSO-*d*_6_) δ (ppm): 8.91 (1H, s), 7.75 (1H, d, *J* = 8.8 Hz), 7.37 (1H, d, *J* = 4.8 Hz), 7.19 (1H, d, *J* = 2.0 Hz), 7.09 (1H, d, *J* = 3.2 Hz), 6.98–6.95 (1H, m), 6.91 (1H, dd, *J*_1_ = 8.8 Hz, *J*_2_ = 2.0 Hz), 6.62 (1H, t, *J* = 6.0 Hz), 4.52 (2H, d, *J* = 6.0 Hz). ^13^C-NMR (125 MHz DMSO-*d*_6_) δ (ppm): 150.1, 147.2, 145.6, 144.2, 135.9, 127.3, 125.6, 125.2, 123.5, 115.1, 102.2, 42.5. HR-MS (ESI^+^) *m/z*: 247.0362 [M+H]^+^, calcd for C_12_H_11_N_2_S_2_^+^, 247.0364.

*Benzothiazol-6-yl-(5-chlorothiophen-2-ylmethyl)-amine* (**3i**). Brown solid, mp: 104–106 °C, 62% yield; ^1^H-NMR (400 MHz, DMSO-*d*_6_) δ (ppm): 8.93 (1H, s), 7.76 (1H, d, *J* = 8.8 Hz), 7.19 (1H, d, *J* = 2.0 Hz), 6.97–6.95 (2H, m), 6.90 (1H, dd, *J*_1_ = 8.8 Hz, *J*_2_ = 2.0 Hz), 6.67 (1H, t, *J* = 6.0 Hz), 4.46 (2H, d, *J* = 6.0 Hz). ^13^C-NMR (125 MHz DMSO-d_6_) δ (ppm): 150.4, 146.9, 145.7, 144.2, 135.9, 126.9, 126.7, 125.3, 123.6, 115.1, 102.4, 42.8. HR-MS (ESI^+^) *m/z*: 280.9980 [M+H]^+^, calcd for C_12_H_10_N_2_S_2_Cl^+^, 280.9974.

*Benzothiazol-6-yl-pyrimidin-5-ylmethyl-amine* **(3j).** Pale yellow solid, mp: 127–129 °C, 51% yield; ^1^H-NMR (500 MHz, DMSO-*d*_6_) δ (ppm): 9.06 (1H, s), 8.91 (1H, s), 8.81 (2H, s), 7.75 (1H, d, *J* = 9.0 Hz), 7.19 (1H, d, *J* = 2.5 Hz), 6.90 (1H, dd, *J*_1_ = 9.0 Hz, *J*_2_ = 2.5 Hz), 6.64 (1H, t, *J* = 6.0 Hz), 4.39 (2H, d, *J* = 6.0 Hz). ^13^C-NMR (125 MHz DMSO-*d*_6_) δ (ppm): 157.6, 156.8 (2C), 150.4, 147.0, 145.7, 136.0, 133.5, 123.7, 115.1, 102.2, 42.5. HR-MS (ESI^+^) *m/z*: 243.0710 [M+H]^+^, calcd for C_12_H_11_N_4_S^+^, 243.0704.

*Anthracen-9-ylmethyl-benzothiazol-6-yl-amine* (**3k**). Yellow solid, mp: 174–176 °C, 64% yield; ^1^H-NMR (400 MHz, DMSO-*d*_6_) δ (ppm): 8.96 (1H, s), 8.66 (1H, s), 8.30 (2H,d, *J* = 8.4 Hz), 8.15–8.13 (2H, m), 7.79 (1H, d, *J* = 8.8 Hz), 7.60–7.52 (5H, m), 7.01 (1H, dd, *J*_1_ = 8.8 Hz, *J*_2_ = 2.0 Hz), 6.32 (1H, t, *J* = 4.0 Hz), 5.14 (2H, d, *J* = 4.0 Hz). ^13^C-NMR (125 MHz DMSO-*d*_6_) δ (ppm): 149.8, 148.2, 145.4, 136.1, 131.6 (2C), 130.7 (2C), 130.2, 129.4 (2C), 127.8, 126.8 (2C), 125.7 (2C), 125.0 (2C), 123.5, 115.1, 101.4, 40.6. HR-MS (ESI^+^) *m/z*: 341.1104 [M+H]^+^, calcd for C_22_H_17_N_2_S^+^, 341.1112.

*Preparation of (E)-(3H-Benzoimidazol-5-yl)-(3-phenyl-allyl)-amine* (**4a**). A mixture of compound **4M** (2 mmol) and *trans*-3-phenylpropenal (2.2 mmol) in ethanol (15 mL) was stirred at room temperature for 8 h, and NaBH_4_ (2.2 mmol) was then added to the solution which was mixed overnight. The solvent concentration was halved by diluting it with water and was extracted by ethyl acetate (3 × 15 mL). The combined organic layers were dried over Na_2_SO_4_ and evaporated. The residue was purified by flash chromatography on silica using a CH_2_Cl_2_/MeOH gradient and the compound 4a was generated in the form of a yellow solid, mp: 140–142 °C, 52% yield; ^1^H-NMR (400 MHz, DMSO-*d*_6_) δ (ppm): 7.90 (1H, s), 7.45–7.29 (6H,m), 7.23–7.20 (1H, m), 6.65–6.61 (3H, m), 6.42 (1H, t, *J* = 5.2 Hz), 3.87 (2H, d, *J* = 5.2 Hz). ^13^C-NMR (125 MHz DMSO-*d*_6_) δ (ppm): 145.7, 139.9, 137.3, 130.4, 129.4, 129.1 (2C), 129.0, 128.0, 127.7, 126.5 (2C), 117.5, 111.5, 94.7, 46.3. HR-MS (ESI^+^) *m/z*: 250.1341 [M+H]^+^, calcd for C_16_H_16_N_3_^+^, 250.1344.

*(3H-Benzoimidazol-5-yl)-(5-methyl-furan-2-ylmethyl)-amine* (**4b**). Compound **4b** was synthesized using a method similar to that of 4a and was isolated as a brown solid. mp: 75–77 °C; 55% yield; ^1^H-NMR (400 MHz; DMSO-*d*_6_) δ (ppm): 8.07 (1H; s); 7.35 (1H; d; *J* = 8.8 Hz); 6.71–6.69 (2H, m); 6.18 (1H, d; *J* = 2.8 Hz); 6.00–5.99 (1H, m); 4.22 (2H; s); 2.25(3H; s). ^13^C-NMR (125 MHz DMSO-*d*_6_) δ (ppm): 152.0; 150.9; 146.8; 139.8; 136.9; 131.5; 117.0; 112.1; 108.1; 106.7; 94.8; 41.3; 13.7. HR-MS (ESI^+^) *m/z*: 228.1135 [M+H]^+^; calcd for C_13_H_14_N_3_O^+^; 228.1137.

### 3.3. Antiviral Assays

Madin-Darby Canine Kidney (MDCK) cells, African green monkey kidney cells (Vero) and Coxsackie viruses (Cox B3 Nancy strain) were all purchased from ATCC. 2.2.15 cells, an HBV-transfected human HepG2 cell line [23], were kindly provided by Y.C. Cheng (Yale Medical School, New Haven, CT, USA). Huh7.5 human liver cells (kindly provided by Vertex Pharmaceuticals, Boston, MA) were cultured in Dulbecco’s Modified Eagle’s Medium (DMEM, Invitrogen, Carlsbad, CA, USA) supplemented with 10% inactivated fetal bovine serum (Invitrogen) and 1% penicillin-streptomycin (Invitrogen). Influenza A strains were all obtained from the Institute of Virology, Chinese Academy of Preventive Medicine. The plasmid pFL-J6/JFH/JC1 containing the full-length chimeric HCV cDNA was kindly provided by Vertex Pharmaceuticals (Boston, MA, USA).

#### 3.3.1. Cytotoxicity Assay

The cytotoxicity of the compounds in MDCK, Vero and HepG2.2.15 cells was monitored *via* cytopathic effect (CPE) assays. The MDCK and Vero cells (2.5 × 10^4^ /well) were plated in a 96-well plate. Following a 24 h hold period, the monolayer of cells was incubated in the presence of various concentrations of the test compounds. Following 48 h of culture at 37 °C under a 5% CO_2_ atmosphere in an incubator, the cells were monitored by CPE. The TC_50_ value was calculated using Reed & Muench analysis.

The HepG2.2.15 cells (2.5 × 10^4^ /well) were plated in a 96-well plate, and after a 48 h incubation, the supernatant was replaced by fresh culture medium containing the test compounds. Cells were cultured for 3 days at 37 °C in a 5% CO_2_ atmosphere in an incubator, and the medium was replaced with fresh medium containing the test compounds for an additional 3 days. The cells were subsequently monitored by CPE and the TC_50_ value was calculated using Reed and Muench analysis.

The Huh7.5 cells (1 × 10^4^ cells/well) were plated in 96-well plates. Six hours later the culture media was replaced with fresh medium containing the test compounds at various concentrations. Cytotoxicity was evaluated with the 3-(4,5-dimethylthiazol-2-yl)-2,5-diphenyltetrazolium bromide (MTT) assay after 96 h. The 50% cytotoxicity concentration (TC_50_) was calculated with Reed and Muench methods.

#### 3.3.2. Anti-Influenza Assays

Confluent MDCK cells grown in 96-well microplates were infected with influenza A at a median tissue culture infective dose TCID_50_ of 100. After 2 h of viral adsorption at 37 °C, the monolayers were washed with PBS and incubated at 37 °C in the maintenance medium with or without different concentrations of test compounds. Viral cytopathic effect (CPE) was measured when the viral control group reached a value of 4 and the antiviral activities of the synthesized compounds were determined by Reed and Muench analyses.

#### 3.3.3. Anti-HBV Assays

The anti-HBV activities of test compounds and a positive control drug were tested in HepG2.2.15 cells. The HepG2.2.15 cells were cultured in 96-well plates and treated with the test compounds at 37 °C for 3 days. The medium was removed, and fresh medium containing the test compounds was added for an additional 3 days. The cells were harvested and intracellular DNA was extracted. The inhibition of viral DNA replication in treated cells *versus* untreated cells was determined by real time PCR.

#### 3.3.4. Anti-HCV Assays

Huh7.5 cells were seeded at a density of 3 × 10^4^ cells/cm^2^. After 24 h of incubation, the cells were infected with an HCV viral stock (approximately 45 IU per cell) and simultaneously treated with Telaprevir, the test compounds, or the solvent control. The culture medium was removed 96 h after inoculation, and the total intracellular RNA and total intracellular proteins were extracted with a Qiagen Kit according to the manufacturer’s instructions. Intracellular HCV RNA was quantified using a one-step real time RT-PCR kit (Invitrogen). Cytotoxicity was tested by the MTT assay, and IC_50_ values were calculated by the Reed and Muench methods.

#### 3.3.5. Anti-Coxsackie B3 Activity Assays

Confluent Vero cells grown in 96-well plates were infected with a median tissue culture infective dose of 100 (100TCID50) Cox B3 viruses. After 1 h of viral adsorption at 37 °C, the monolayers were washed with phosphate buffered saline (PBS) and incubated at 37 °C in the maintenance medium (MEM plus 2% fetal bovine serum (FBS)) with or without different concentrations of test compounds. Viral cytopathic effect (CPE) was measured when the viral control group reached a level of 4 and the antiviral activity of test compounds was determined by the Reed and Muench analyses.

## 4. Conclusions

A total of 24 novel benzo-heterocyclic amine derivatives were designed, synthesized and screened for their antiviral activities towards influenza A, HBV, HCV and Cox B3 in this study. In general, the target compounds were more effective against RNA viruses (influenza A, HCV and Cox B3) than the DNA virus (HBV), and the majority of the synthesized compounds simultaneously exhibited potent activity against the influenza A, HCV and Cox B3 viruses. Additionally, a similar trend was observed among the SARs of the synthesized compounds towards the RNA virus. Different substituents on the aromatic ring had certain effects on the activity. For example, an electron-withdrawing substituent on the aromatic or heteroaromatic ring favored antiviral activity. Notably, compounds **3f** and **3g** showed potent antiviral activity towards the entire virus panel tested at low micromolar concentrations, especially compound **3f** which was active against the entire virus panel tested with an IC_50_ value of approximately 5.0 μM. The detailed structure optimization of compound **3f** and the study of the mechanisms of action of these compounds are ongoing in our laboratory.

## References

[1] Zhang X., Qi X., Zhang Q., Zeng X., Shi Z., Jin Q., Zhan F., Xu Y., Liu Z., Feng Z. (2013). Human 4F5 single-chain Fv antibody recognizing a conserved HA1epitope has broad neutralizing potency against H5N1 influenza A viruses of different clades. Antivir. Res..

[2] Metersky M.L., Masterton R.G., Lode H., File T.M., Babinchak T. (2012). Epidemiology, microbiology, and treatment considerations for bacterial pneumonia complicating influenza. Int. J. Infect. Dis..

[3] Choo Q.L., Kuo G., Weiner A.J., Overby L.R., Bradley D.W., Houghton M. (1989). Isolation of a cDNA clone derived from a blood-bornc non-A, non-B viral hepatitis genomc. Science.

[4] Alter M.J. (2007). Epidemiology of hepatitis C virus infection. World J. Gastroenterol..

[5] Kesel A.J. (2011). Broad-spectrum antiviral activity including human immunodeficiency and hepatitis C viruses mediated by a novel retinoid thiosemicarbazone derivative. Eur. J. Med. Chem..

[6] Lawitz E., Sulkowski M., Jacobson I., Kraft W.K., Maliakkal B., Al-lbrahim M., Gordon S.C., Kwo P., Rockstroh J.K., Panorchan P. (2013). Characterization of vaniprevir, a hepatitis C virus NS3/4A protease inhibitor, in patients with HCV genotype 1 infection: Safety, antiviral activity, resistance, and pharmacokinetics. Antivir. Res..

[7] Huang C.C., Kuo T.M., Yeh C.T., Hu C.P., Chen Y.L., Tsai Y.L., Chen M.L., Chou Y.C., Chang C. (2013). One single nucleotide difference alters the differential expression of spliced RNAs between HBV genotypes A and D. Virus Res..

[8] Pinkert S., Klingel K., Lindig V., Dörner A., Zeichhardt H., Spiller O.B., Fechner H. (2011). Virus-host coevolution in a persistently coxsackievirus B3-infected cardiomyocyte cell line. J. Virol..

[9] Bedard K.M., Wang M.L., Proll S.C., Loo Y.M., Katze M.G., Gale M., Ladonato S.P. (2012). Isoflavone agonists of IRF-3 dependent signaling have antiviral activity against RNA viruses. J. Virol..

[10] Richman D.D. (2006). Antiviral drug resistance. Antivir. Res..

[11] Colman P.M. (2009). New antivirals and drug resistance. Annu. Rev. Biochem..

[12] Krepstakies M., Luciflra J., Naqel C.H., Zeisel M.B., Holstermann B., Hohenberq H., Kowalski I., Gutsmann T., Baumert T.F., Brandenburg K. (2012). A new class of synthetic peptide inhibitors blocks attachment and entry of human pathogenic viruses. J. Infect. Dis..

[13] ElSawy K.M., Twarock R., Verma C.S., Caves L.S. (2012). Peptide inhibitors of viral assembly: A novel route to broad-spectrum antivirals. J. Chem. Inform. Model..

[14] Zhong Z.J., Zhang D.J., Peng Z.G., Li Y.H., Shan G.Z., Zuo L.M., Wu L.T., Li S.Y., Gao R.M., Li Z.R. (2013). Synthesis and antiviral activity of a novel class of (5-oxazolyl)phenyl amines. Eur. J. Med. Chem..

[15] Wang T., Zhang Z., Wallace O.B., Deshpande M., Fang H., Yang Z., Zadjura L.M., Tweedie D.L., Huang S., Zhao F. (2003). Discovery of 4-benzoyl-1-[(4-methoxy-1H- pyrrolo[2,3-b]pyridin-3-yl)oxoacetyl]-2- (R)-methylpiperazine (BMS-378806): a novel HIV-1 attachment inhibitor that interferes with CD4-gp120 interactions. J. Med. Chem..

[16] Garuti L., Roberti M., de Clercq E. (2002). Synthesis and antiviral/antiproliferative activity of some N-sulphonylbenzimidazoles. Bioorg. Med. Chem. Lett..

[17] Cheng J., Xie J., Luo X. (2005). Synthesis and antiviral activity against Coxsackie virus B3 of some novel benzimidazole derivatives. Bioorg. Med. Chem. Lett..

[18] De Sá Alves F.R., Barreiro E.J., Fraga C.A. (2009). From nature to drug discovery: The indole scaffold as a ‘privileged structure’. Mini Rev. Med. Chem..

[19] Yee Y.K., Bernstein P.R., Adams E.J., Brown F.J., Cronk L.A., Hebbel K.C., Vacek E.P., Krell R.D., Snyder D.W. (1990). A novel series of selective leukotriene antagonists: Exploration and optimization of the acidic region in 1,6-disubstituted indoles and indazoles. J. Med. Chem..

[20] Jagadeesh R.V., Wienhöfer G., Westerhaus F.A., Surkus A.E., Pohl M.M., Junqe H., Junqe K., Beller M. (2011). Efficient and highlyselectiveiron-catalyzedreduction of nitroarenes. Chem. Commun..

[21] Hrobárik P., Hrobáriková V., Sigmundová I., Zahradník P., Fakis M., Polyzos I., Persephonis P. (2011). Benzothiazoles with tunableelectron-withdrawingstrength and reverse polarity: A route to triphenylamine-based chromophores with enhanced two-photon absorption. J. Org. Chem..

[22] Rahaim R.J., Maleczka R.E. (2005). Pd-catalyzed silicon hydride reductions of aromatic and aliphatic nitro groups. Org. Lett..

[23] Sells M.A., Chen M.L., Acs G. (1987). Production of hepatitis B virus particles in Hep G2 cells transfected with cloned Hepatitis B virus DNA. Proc. Natl. Acad. Sci. USA..

